# Evaluation and analysis of the impact of seamless nursing on angina control, disease prognosis, and nursing quality in older adult patients with coronary heart disease

**DOI:** 10.3389/fcvm.2025.1560960

**Published:** 2025-10-07

**Authors:** Fugui Wei, Yali Wang, Wei Han

**Affiliations:** Department of Cardiovascular Medicine, The Zhejiang Hospital, Hangzhou, Zhejiang, China

**Keywords:** seamless nursing, coronary heart disease, angina, adverse events, nursing quality

## Abstract

**Objective:**

To investigate the impact of seamless nursing on angina control, disease prognosis, and nursing quality in older adult patients with coronary heart disease (CHD).

**Methods:**

A total of 100 CHD patients who were hospitalized in the cardiology department of our hospital from February 2024 to January 2025 were randomly divided into a control group (50 patients) and a study group (50 patients) using a random number table. The control group received conventional nursing care, while the study group received seamless nursing care. The Seattle Angina Questionnaire (SAQ) scores, incidence of adverse events, and nursing quality scores of both groups were observed and compared.

**Results:**

The SAQ scores of the study group were significantly higher than those of the control group, with improvements in physical activity limitations, angina frequency, and angina stability (*P* < 0.05). The incidence of adverse events in the study group was significantly lower than that in the control group (*P* < 0.05). The nursing quality scores for service capability, operational norms, humanistic care, and health education in the study group were significantly higher than those in the control group, with a higher total score (*P* < 0.05).

**Conclusion:**

Seamless nursing can effectively reduce the frequency of angina, alleviate physical activity limitations, decrease the incidence of adverse events, and improve patients’ quality of life in older adult patients with coronary heart disease. It also appears to improves nursing workflows and enhances nursing quality, suggesting potential value for further clinical exploration in larger studies.

## Background

Coronary heart disease (CHD) is a prevalent, multifactorial condition primarily affecting older adults. It is characterized by the progressive narrowing of coronary arteries due to atherosclerosis, which restricts the heart's blood supply. This leads to myocardial hypoxia, ischemia, and, in severe cases, myocardial necrosis. Clinically, CHD often manifests as recurrent chest pain, typically presenting as angina pectoris or a sensation of chest pressure, accompanied by general discomfort. In more critical cases, CHD can precipitate acute events such as myocardial infarction, heart failure, or cardiogenic shock, posing significant risks to a patient's life and overall well-being (1).

CHD is more prevalent in men over 40, but rising living standards have led to a significant increase in cases, establishing CHD as a major global public health concern (2). Consequently, optimizing treatment regimens and nursing strategies has become increasingly critical. While traditional approaches primarily emphasize pharmacological management, they often fail to address the multifaceted nature of the disease. This has spurred growing interest in innovative care models designed to enhance patient outcomes and quality of life.

To address the complex demands of CHD management, seamless care, a patient-centered model, has been increasingly adopted in clinical practice. This approach prioritizes uninterrupted continuity and coordination across the patient's healthcare journey, from admission to discharge. By integrating multidisciplinary care, encompassing medical, nutritional, psychological, and rehabilitative support, seamless care delivers a comprehensive strategy that addresses both physical and emotional needs (3). The model ensures consistent, cohesive care, boosting patient satisfaction and promoting efficient recovery. Clinical studies and patient outcomes confirm that seamless care reduces hospitalization durations, strengthens nurse-patient relationships, and enhances overall hospital service quality and patient satisfaction (4, 5).

This study aims to explore the application and effectiveness of seamless care in older adult patients with CHD. It specifically evaluates the model's impact on managing angina pectoris, improving disease prognosis, and elevating the quality of nursing care. By integrating seamless care into CHD management, the research aims to assess its potential to reduce the frequency and severity of chest pain, enhance patients’ physical activity tolerance, and decrease the incidence of adverse cardiovascular events.

## Materials and methods

### Study design and general information

This prospective study utilized a convenience sampling method to select 100 patients diagnosed with coronary artery disease who were admitted for inpatient treatment in the cardiovascular department of our hospital between February 2024 to January 2025. To minimize selection bias, patients were randomly assigned to either the control group or the observation group, using a random number table, with 50 patients in each group.

Inclusion Criteria: (1) Patients were diagnosed with coronary artery disease and aged 65 years or older. (2) Patients had normal cognitive function and were able to communicate verbally. (3) Disease duration was greater than 3 months. (4) Patients voluntarily agreed to participate in the study.

Exclusion Criteria: (1) Patients with New York Heart Association (NYHA) Class IV heart failure. (2) Patients with severe concurrent organ diseases, such as those affecting the brain, liver, lungs, or kidneys. (3) Patients already involved in similar studies. (4) Patients lost to follow-up during the study.

**Figure 1:**
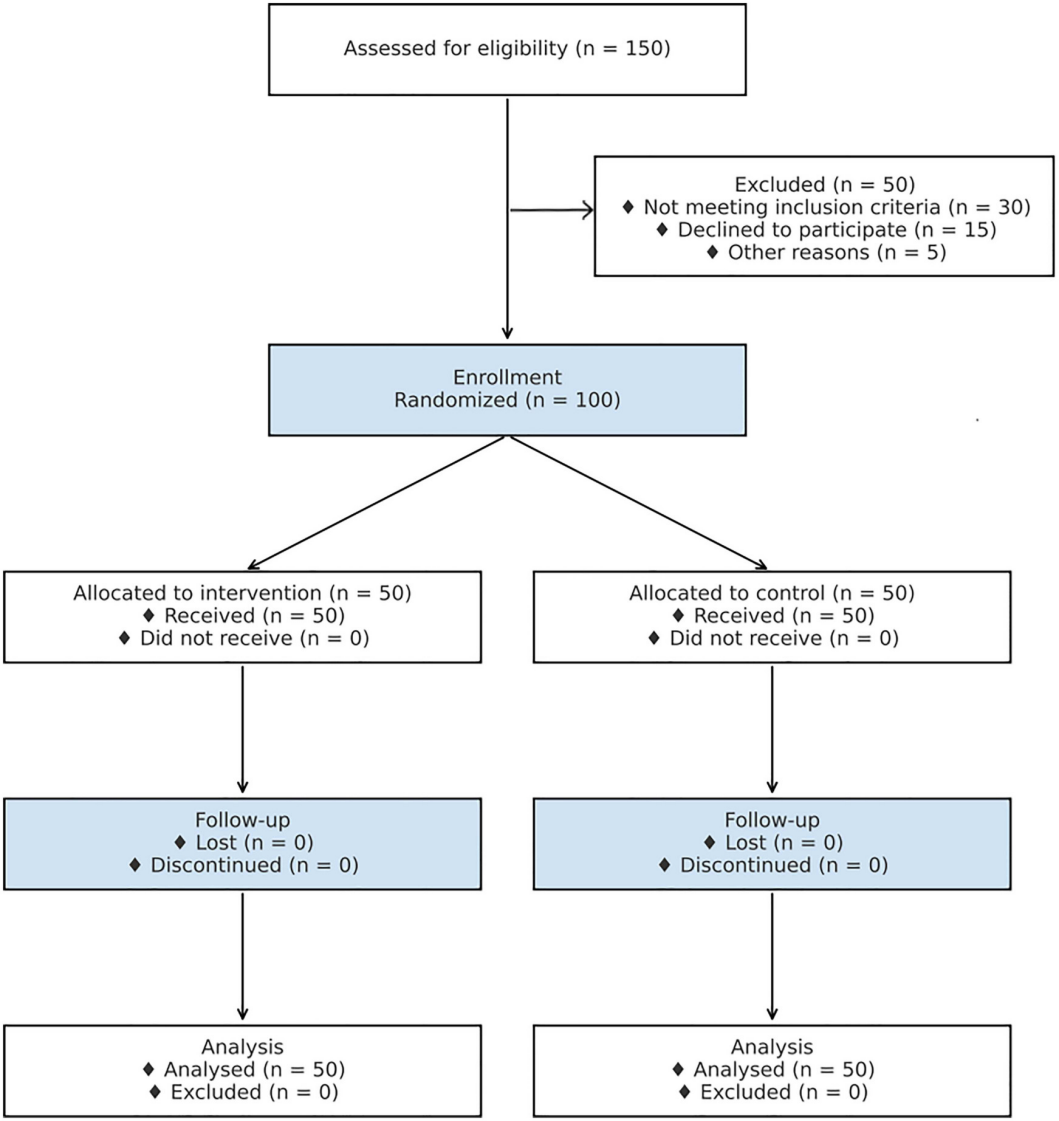
CONSORT flow diagram of participant enrollment, allocation, follow-up, and analysis.

This study was conducted in compliance with ethical guidelines set by the institutional review board (IRB). Informed consent was obtained from all participants prior to their inclusion in the study, and all data were anonymized to protect patient confidentiality. A CONSORT flow diagram is provided in Figure 1 to illustrate participant enrollment, allocation, follow-up, and analysis.

## Methods

The control group received conventional nursing care, which included standard practices such as admission education, routine ward rounds, dietary management, and other general nursing procedures. In contrast, the observation group was provided with a comprehensive seamless care model, in addition to the routine treatments, as outlined below:

A seamless nursing team was formed, consisting of a team leader and 2–3 team members per group. The nursing team for this study consisted of 25 nurses, including three with senior professional titles, five charge nurses, ten staff nurses, and seven auxiliary nursing staff. The average age of the nurses was (32.75 ± 4.31) years, with a range from 21 to 49 years. The nurses’ length of service ranged from 1 to 20 years, with a mean of (5.94 ± 1.75) years. The team leader was responsible for developing and implementing the overall care plan for each patient, ensuring its execution throughout the patient's hospitalization. Additionally, the team leader was tasked with overseeing quality control, coordinating care with physicians, and ensuring that the care plan was adhered to at every stage. Each team member, in collaboration with the responsible physician, was assigned to no more than three patients, ensuring personalized and focused care. This smaller patient-to-nurse ratio allowed the team to deliver individualized attention, thereby improving the quality of care. Upon admission, both patients and their families were introduced to their healthcare team, including physicians and nurses, to ensure complete transparency and foster continuous, uninterrupted communication. This initial introduction was key in setting the foundation for collaborative, high-quality care and aimed to reduce potential barriers to communication, thus enhancing the patient's overall experience and satisfaction.

To further enhance the continuity of care, seamless care was organized on a 24-hour shift system. This ensured that nursing staff were always available, providing around-the-clock support. A critical component of this system was the scheduling of shifts, which was managed by the team leader. Every Monday, a meeting was held to organize and arrange the shift schedule for the upcoming week, ensuring a balanced workload and preventing fatigue among staff. This was essential not only for maintaining the physical and mental well-being of the nursing team but also for minimizing the risks associated with staff exhaustion, such as reduced attentiveness and compromised care quality. The goal was to maintain a well-rested, attentive nursing staff, capable of providing uninterrupted, comprehensive care across all shifts—typically 8-hour day shifts and continuous overnight monitoring.

Before patient admission, the team leader organized a preparatory meeting with the nursing team and the attending physician. This meeting ensured that all team members were fully informed about the patient's medical history, current condition, treatment plans, test results, and any allergies. By thoroughly reviewing this information prior to the patient's arrival, the nursing staff was equipped to deliver precise, personalized, and timely care. This proactive approach allowed the team to anticipate the patient's needs and tailor interventions accordingly, ensuring optimal care delivery from the very start of the hospitalization.

Upon arrival, the team leader personally greeted the patient and introduced the assigned nursing staff. With consent, the team established contact via digital platforms, such as WeChat (Tencent Holdings Limited, Shenzhen, China), to enhance communication. This enabled continuous updates on medical orders, treatment plans, meal schedules, and test results. Sharing daily updates with families improved transparency, reduced misunderstandings, built trust, facilitated cooperation, and contributed to positive experiences and potentially shorter treatment durations.

In addition to the standard nursing care, the seamless care team took an active role in observing and adjusting to the patient's personal habits, such as sleep patterns, eating behaviors, and emotional well-being. Nurses were trained to make subtle yet impactful adjustments to the patient's environment, such as optimizing lighting to support circadian rhythms and regulate sleep patterns, which could improve the patient's overall health and cooperation with the treatment plan. The team also provided tailored advice on diet and lifestyle changes, guiding patients towards healthier habits and suggesting individualized meal plans to meet their specific health needs. For eligible patients, the nursing staff recommended light physical activity and exercises to help them achieve a balanced physical and mental state, promoting active participation in their treatment plan and enhancing their overall well-being.

Furthermore, regular communication with patients was encouraged to improve their understanding of their condition, thereby fostering a more positive attitude towards treatment and compliance with prescribed therapies. This proactive communication strategy aimed to empower patients, reduce anxiety, and improve their engagement in the rehabilitation process.

After discharge, the nursing team continued to support the patient through follow-up communication. Nurses kept in touch with patients to inquire about their daily activities, offering further guidance on diet, lifestyle modifications, and rehabilitation exercises. This ongoing support ensured that patients continued to adhere to their post-discharge care plan, promoting sustained recovery and minimizing the risk of readmission. Additionally, health information was disseminated to the broader public via official hospital channels, such as WeChat (Tencent Holdings Limited, Shenzhen, China). This not only supported the patients but also served to raise awareness about coronary heart disease, thereby enhancing the hospital's public image and reputation within the community.

## Observation indicators

### Seattle angina questionnaire (SAQ)

The Seattle Angina Questionnaire (SAQ) is a well-established, disease-specific instrument designed to evaluate the functional status and quality of life in patients with coronary artery disease (CAD). In this study, the SAQ was utilized to assess the physical functioning and quality of life in 100 patients diagnosed with CAD. The SAQ comprises five major domains with 19 items: Physical Limitation (Item 1), Angina Stability (Item 2), Frequency of Angina Attacks (Items 3–4), Satisfaction with Treatment (Items 5–8), and Understanding of Disease (Items 9–11). Each item is rated individually, and scores for each domain range from 0 to 100, with higher scores reflecting better health outcomes, including improved quality of life and physical functioning. This study specifically focused on three domains: Physical Activity Limitation, Frequency of Angina Attacks, and Angina Stability. The SAQ was administered by trained nurses, with higher scores indicating better patient outcomes. The tool has demonstrated excellent reliability and validity in numerous clinical settings, ensuring its robustness as a measure of disease severity and treatment impact in CAD patients (6).

### Adverse event incidence

The incidence of adverse events was monitored for a period of six months following the intervention. Adverse events were defined as follows: recurrent angina (new episodes of chest pain requiring medical intervention, confirmed per ACC/AHA guidelines (7); myocardial infarction (confirmed by electrocardiogram, cardiac enzymes, and clinical symptoms (8); and all-cause mortality (death from any cause (9). These definitions reference standard outcome measures in cardiovascular disease trials to ensure consistency and comparability. Ascertainment was performed through structured follow-up by designated nurses via phone calls or home visits every month, with events verified against medical records. Independent adjudication was conducted by a cardiologist not involved in the intervention to minimize bias. This follow-up strategy ensured that patients’ health status was actively monitored, and any complications or new health issues were promptly identified and addressed.

### Nursing quality evaluation

Nursing quality was evaluated using an institution-specific nursing quality assessment form, which assessed four key aspects of nursing care: service capabilities, compliance with procedural standards, humanistic care, and health education. Before patient discharge, the nurse manager performed the evaluation, assigning scores for each category. Each aspect was rated on a 25-point scale, with a maximum possible score of 100 points. Higher scores were indicative of superior nursing quality, reflecting more effective patient care. The internal consistency of the nursing quality scale was validated through the Cronbach's *α* coefficient, which exceeded 0.7, indicating reliable and consistent measurement of the nursing quality parameters.

### Data collection methods

At enrollment, participants were asked to complete a basic demographic survey to collect pertinent background information. Following the intervention, the SAQ questionnaire was administered to assess the patients’ quality of life and physical functioning. Trained nurses explained the study's objectives, the structure of the questionnaire, the completion process, and the time required to fill it out. In cases where patients had difficulty completing the questionnaire independently, nurses assisted them by asking the questions directly and recording their responses. All questionnaires were collected on-site immediately after they were completed. A total of 100 questionnaires were distributed, and 100 valid responses were returned, resulting in a 100% response rate. This ensured a high level of data completeness and reliability.

### Statistical methods

Statistical analysis was performed using SPSS 26.0 software (IBM Corp., Armonk, NY, USA). Continuous data are presented as mean ± standard deviation (SD) and were compared between the two groups using independent samples t-tests. Categorical data are expressed as frequencies (percentages) and were analyzed using chi-square (*χ*²) tests. For binary endpoints, we reported risk ratios (RRs) with 95% confidence intervals using the Katz log method, and compared proportions with Pearson's chi-square test (without continuity correction for the primary analysis), with Fisher's exact test as a sensitivity analysis in case of sparse cells. For sensitivity analyses, we (i) excluded deaths from the composite endpoint and (ii) repeated per-protocol analyses; the latter was identical to the intention-to-treat analysis because there was no crossover and no loss to follow-up. For continuous outcomes (nursing quality totals and SAQ domains), we used Welch's *t*-test and reported mean differences with 95% CIs. Two-sided *p* < 0.05 was considered statistically significant. No adjustments were made for multiple comparisons due to the exploratory nature of the study.

## Results

### Baseline characteristics

This study enrolled 100 patients with coronary artery disease, randomly assigned to a control group of 31 males and 19 females with a mean age of 66.73 ± 4.61 years, disease duration of 8.39 ± 4.27 years, and weight of 63.44 ± 6.26 kg, or an intervention group of 30 males and 20 females with a mean age of 66.52 ± 4.48 years, disease duration of 8.21 ± 4.15 years, and weight of 63.18 ± 6.12 kg, showing no significant baseline differences, *P* > 0.05. Baseline disease characteristics included stable angina at 60% in the control group and 62% in the intervention group, NSTEMI at 25% in the control group and 24% in the intervention group, and STEMI at 15% in the control group and 14% in the intervention group, with coexisting conditions such as hypertension at 72% in the control group and 70% in the intervention group, and diabetes mellitus at 48% in the control group and 46% in the intervention group, showing no significant differences, *P* > 0.05. Results are summarized in Table 1.

**Table 1:** Baseline characteristics of the study groups.

Characteristic	Control group (*n* = 50)	Intervention group (*n* = 50)	Statistic (t/*χ*²)	*P*-value
Age (years, mean ± SD)	66.73 ± 4.61	67.12 ± 4.89	t = 0.410	0.682
Sex [male, *n* (%)]	31 (62.00)	30 (60.00)	χ² = 0.042	0.838
Body weight (kg, mean ± SD)	63.44 ± 6.26	63.18 ± 6.12	t = 0.210	0.834
Disease duration (years, x¯ ± s)	8.39 ± 4.27	8.21 ± 4.15	t = 0.214	0.831
Clinical presentation [*n* (%)]			χ² = 0.342	0.843
Stable angina	30 (60.00)	31 (62.00)		
NSTEMI	12 (24.00)	13 (26.00)		
STEMI	8 (16.00)	6 (12.00)		
Comorbidities (%)				
Hypertension [*n* (%)]	36 (72.00)	35 (70.00)	χ² = 0.049	0.826
Diabetes mellitus [*n* (%)]	24 (48.00)	23 (46.00)	χ² = 0.040	0.841

### Comparison of SAQ

After the intervention, the intervention group showed significantly higher scores in physical activity limitation, frequency of angina attacks, and stability of angina compared to the control group. These differences were statistically significant (*P* < 0.05). The results are summarized in Table 2.

**Table 2:** Comparison of Seattle angina questionnaire scores between groups.

Group	*N*	Physical activity limitation	Frequency of angina attacks	Stability of angina
Control group	50	71.35 ± 5.44	56.24 ± 5.01	81.69 ± 6.33
Intervention group	50	81.27 ± 6.56	65.41 ± 5.72	90.62 ± 5.03
t-value		8.231	8.528	7.810
*P*-value		<0.001	<0.001	<0.001

### Comparison of adverse event incidence

The incidence of adverse events was significantly lower in the intervention group compared with the control group (*P* < 0.05). Specifically, the composite endpoint occurred in 12% (6/50) of patients in the intervention group vs. 28% (14/50) in the control group (RR 0.43, 95% CI 0.18–1.03; *P* = 0.046) (Table 3). Individual adverse events were infrequent but consistently favored the intervention group, including recurrent angina (8% vs. 16%, RR 0.50, 95% CI 0.16–1.55), myocardial infarction (4% vs. 10%, RR 0.40, 95% CI 0.08–1.97), and all-cause death (0% vs. 2%, RR 0.33, 95% CI 0.01–7.99) (Table 4). A sensitivity analysis excluding deaths from the composite endpoint showed a similar trend (RR 0.46, 95% CI 0.19–1.12; *p* = 0.125) (Table 5).

**Table 3:** Comparison of adverse event incidence between groups.

Group	*N*	Recurrent angina (%)	Myocardial infarction (%)	All-cause mortality (%)	Total adverse event incidence (%)
Control group	50	8 (16.00)	5 (10.00)	1 (2.00)	14 (28.00)
Intervention group	50	4 (8.00)	2 (4.00)	0 (0)	6 (12.00)
*χ*^2^-value					4.000
*P*-value					0.046

**Table 4:** Relative risks and 95% confidence intervals for individual components of adverse events between groups.

Endpoint	Intervention (*n/N*)	Control (*n*/*N*)	RR (95% CI)	*P*-value
Composite	6/50	14/50	0.43 (0.18–1.03)	0.078
Recurrent angina	4/50	8/50	0.50 (0.16–1.55)	0.357
Myocardial infarction	2/50	5/50	0.40 (0.08–1.97)	0.436
All-cause death	0/50	1/50	0.33 (0.01–7.99)	1.000

Values are *n*/*N* unless otherwise indicated. RR 95% CIs by Katz log method; Fisher's exact *p* shown for sparse counts.

**Table 5:** Sensitivity analysis.

Endpoint	Intervention (*n*/*N*)	Control (*n*/*N*)	RR (95% CI)	*P*-value
Composite (excluding death)	6/50	13/50	0.46 (0.19–1.12)	0.125

### Comparison of nursing quality scores

The intervention group scored significantly higher than the control group in all four nursing quality domains: service capability, procedural compliance, humanistic care, and health education. The total nursing quality score in the intervention group was also significantly higher than in the control group. These differences were statistically significant (*P* < 0.05). The results are summarized in Table 6.

**Table 6:** Comparison of nursing quality scores between groups.

Group	Service capability	Procedural compliance	Humanistic care	Health education	Total score
Control group	18.33 ± 1.15	18.45 ± 0.97	18.01 ± 0.95	18.43 ± 1.32	78.25 ± 5.98
Intervention group	21.17 ± 2.01	21.38 ± 1.35	21.88 ± 1.13	20.71 ± 1.25	87.37 ± 6.39
t-value	6.132	8.813	13.107	6.271	5.210
*P*-value	<0.001	<0.001	<0.001	<0.001	<0.001

## Discussion

CHD is a prevalent cardiovascular condition primarily caused by coronary artery atherosclerosis, which leads to a reduced blood supply to the heart. The disease is associated with high morbidity and mortality rates, posing a significant threat to both patient safety and quality of life (10). Among patients with CHD, angina pectoris is one of the most common and debilitating symptoms. Angina is characterized by recurrent episodes of chest pain, which not only severely affect the patient's quality of life but also pose a fatal risk to their health. In many cases, angina can precipitate other serious cardiovascular events, further complicating the patient's condition (11–13).

In this single-centre randomized study of 100 coronary heart disease patients, baseline characteristics were balanced between groups. Compared to usual care, the intervention improved health status on the Seattle Angina Questionnaire, enhancing physical limitation, angina frequency, and stability domains. The intervention also reduced the primary composite adverse event rate and increased nursing-quality scores compared to controls. A sensitivity analysis excluding deaths supported these findings.

Although the composite endpoint showed a borderline significant reduction, individual components were rare and underpowered to show between-group differences. The composite was more sensitive to overall differences in adverse outcomes. These findings, together with the single-centre design and lack of independent adjudication, warrant cautious interpretation and external validation in multicentre, adequately powered, prospectively registered trials.

Despite advances in pharmacological management, CHD remains a chronic condition often requiring lifelong treatment and comprehensive care approaches to optimize outcomes (14). As a result, patients often experience negative emotions, including frustration or resistance to long-term medication regimens. Given these challenges, it is essential to focus on effective clinical management strategies that prioritize symptom control and enhance quality of life. Specifically, the effective control of angina is crucial for improving patient prognosis and plays an indispensable role in managing disease progression (15).

Seamless nursing is an emerging patient-centered nursing model designed to maximize the benefits for patients by focusing on the continuity and quality of care. This model emphasizes identifying and eliminating gaps in care, with the goal of improving the overall healthcare experience for patients (16–18). In CHD treatment, seamless nursing enables for 24-hour continuous monitoring of angina and other symptoms, facilitating early detection of complications and prevention of emergencies. By addressing issues promptly, this approach significantly reduces acute cardiovascular events.

Moreover, seamless nursing not only addresses the immediate medical needs of patients but also enhances their physical and mental well-being. By integrating psychological support, this model effectively reduces the incidence of angina episodes triggered by psychological stressors. Additionally, seamless care has been shown to improve patients’ self-immune responses, contributing to better overall health outcomes. By monitoring and encouraging lifestyle improvements, such as diet and exercise, it helps maintain favorable health post-discharge. This extends to follow-up guidance, aiding long-term management. While low patient-nurse ratios enhance personalization, they may increase costs. Mitigation strategies include telehealth integration, efficient training, and cost-benefit analyses; long-term reductions in readmissions and events can offset expenses, supporting viability in resource-constrained settings (19).

Seamless nursing also promotes stronger communication with patients’ families. By educating family members on important aspects of CHD management, including dietary recommendations, lifestyle modifications, contraindications, and overall care, families are better equipped to support patients both during and after hospitalization. This fosters a collaborative approach to care and enhances patient compliance with post-discharge treatment plans, ultimately reducing the risk of recurrent angina episodes. Furthermore, it strengthens provider-patient relationships, leading to greater satisfaction and adherence, improving nursing quality and hospital reputation.

In conclusion, seamless nursing demonstrates potential in reducing angina incidence, alleviating activity limitations, and lowering adverse events among older adult CHD patients within our setting. This approach also shows promise in enhancing quality of life, improving nursing workflows, and fostering collaboration among patients, families, and providers. However, these preliminary findings from a small, single-center study suggest the need for validation through larger, multicenter randomized controlled trials to assess its broader applicability and long-term effects before considering widespread clinical adoption.

## Data Availability

The original contributions presented in the study are included in the article/Supplementary Material, further inquiries can be directed to the corresponding author.
